# Nursing students’ metaphors of first clinical experiences of encountering patients with mental disorders

**DOI:** 10.1186/s12912-024-01780-9

**Published:** 2024-02-04

**Authors:** Fatemeh Mansouri, Azar Darvishpour

**Affiliations:** 1 Department of Nursing, Zeyinab (P.B.U.H) School of Nursing and Midwifery, Guilan University of Medical Sciences, Rasht, Iran; 2 Social Determinants of Health (SDH) Research Center, Guilan University of Medical Sciences, Rasht, Iran

**Keywords:** Nursing, Students, Mental disorders, Metaphor

## Abstract

**Background:**

Psychiatric wards are one of the most stressful medical centers. Apprenticeship in mental health can cause feelings of stress and anxiety among nursing students. Investigating nursing students’ beliefs about mental illnesses is very important to improve nursing education. The present study aimed to identify nursing students’ metaphors for their first clinical experiences of encountering patients with mental disorders.

**Methods:**

This descriptive qualitative study was conducted on 18 undergraduate nursing students studying in two nursing colleges at Guilan University of Medical Sciences, in the north of Iran, in 2022. The data were collected through semi-structured interviews and analyzed using Critical Metaphor Analysis by the MAXQDA 2007 software.

**Results:**

The analysis of nursing students’ metaphors led to the emergence of 36 metaphors and 5 categories. These categories were “experience of dealing with a mental patient is similar to fear mixed with excitement”, " patient is similar to an errant human”, " psychiatric hospital is similar to a prison”, “nurse is similar to a prison guard”, and “clinical instructor is similar to a supporter, sympathetic and knowledgeable friend”.

**Conclusions:**

The results showed their negative attitude towards the psychiatric hospital and health care providers. It is suggested that the findings of this study be taken into consideration in the planning of clinical education of nursing students.

**Supplementary Information:**

The online version contains supplementary material available at 10.1186/s12912-024-01780-9.

## Background

Psychiatric disorders are one of the main and important public health issues around the world. These disorders are associated with a huge personal, social, and economic burden and affect different areas of a person’s life [1–2]. Negative perceptions, attitudes, and beliefs about mental illness not only affect people with these illnesses but also make those who are tasked with helping and caring for them avoid doing so for fear of being harmed. Lack of understanding of the real structure of mental illnesses due to judgments caused by false beliefs, fears, and negative attitudes towards these diseases is considered a big problem and makes it difficult to help and care for people with mental illnesses [3]. Studies show that health professionals use definitions such as dangerous and unpredictable for patients with mental disorders, although they do so to a lesser extent than the public [4–5]. Negative attitudes toward mental illness by Health Care Professionals can have adverse consequences on people with mental illness from delays in seeking help to decreased quality of care provided [6].

Psychiatric nursing is a branch of nursing that serves people who suffer from psychiatric disorders by combining educational learning and applying the principles of human communication and nursing theories, as well as by acquiring sufficient skills and expertise for nursing care [7]. It is a unique set of knowledge and skills that uses a comprehensive approach to health and well-being with the participation of individuals, families, and communities [8]. The psychiatric wards are one of the most stressful centers among medical and educational centers, and nursing in these wards is considered a stressful occupation due to the type of patients and the problem of communicating with them. Communicating with patients, the heavy workload, and the heavy responsibility of caring for specific patients suffering from psychological problems are among the stress factors of this profession [9]. Clinical education is an important part of nursing education and has a significant impact on the preparation of nursing graduates for the nursing profession [10]. Clinical education has multiple goals, including promoting reflection and critical thinking, promoting inter-professional learning, and promoting the development of students’ clinical reasoning skills, as well as supporting the educational role of teachers [11].

In Iran, the BSN program is the first level of academic nursing education. Nursing students are selected centrally from among the volunteers of the experimental group and through the national entrance exam [12]. Similar to most countries, the Iranian BSN is a four-year program with 8 semesters. Each academic year in Iran consists of two semesters: the first semester begins in late September and ends in late January, and the second semester begins in February and ends in June. On the contrary to other countries, all Iranian universities must follow a basic curriculum established by the Ministry of Health and Medical Education (MOHME), although some minor flexibility is allowed within the predetermined curriculum [13]. The clinical training process in Iran is arranged from simple to difficult and takes place during patients’ care based on the nursing process. Students begin their clinical training from the second semester and this is run concurrently with theoretical courses until the end of the third year. In the fourth year, students participate in full-time hospital-based education apprenticeships. During clinical education, students can work with patients in various departments of general hospitals (internal-surgical, pediatric, obstetrics and gynecology, psychiatry, emergency, and critical care) [14]. Nursing students take a theoretical course in social and personal psychology (worth two credits), mental health nursing I and II, and a practicum in mental health nursing (worth two credits). The Mental Health Nursing (MHN) course is offered to third-year BSN students, by the national nursing curriculum. The MHN is a six-credit hour course, three clinical (153 h) and three theory (51 h) [15].

Because of the characteristics of psychiatric disorders, it is difficult for students to understand and interact with patients suffering from psychiatric disorders [16]. Among all clinical training environments in nursing, training in mental wards may be one of the most difficult experiences [17]. Even if the students understood the concepts and knowledge acquired through textbooks, they would still feel extremely uneasy and helpless when caring for these patients in clinical practice due to inadequate professional training [16]. Undergraduate students often enter the mental health course with a preconceived image of patients that is influenced by the media and a lack of proper knowledge about the patients’ conditions, which is exacerbated by the attitudes of their families. Therefore, students may experience tension, fear, or high levels of stress and anxiety [17]. This issue can lead to negative experiences and attitudes toward mental health nursing, consequently preventing them from having a comfortable and informative experience [18], and even leading them to avoid choosing psychiatric nursing as a career [17]. Currently, there is limited evidence of nursing students’ attitudes toward mental illnesses [17–20] and this is while analyzing how nursing students understand mental illnesses and examining their views and recommendations about these illnesses and stigmatization is very important for improving nursing education in the future [21].

On the other hand, from the student-centered education perspective, the top role of teachers involves facilitating students’ learning, creating a democratic learning environment, and helping students’ total development, especially that of their habits of mind. A major responsibility of teachers is to investigate what is happening in the minds of students and how they learn. In this model, learning and teaching processes are largely based on existing experiences [22]. Empowering learners contributes to the development of positive feelings and attitudes and to the establishment of safe environments, where learners are respected and where emotions can be safely addressed [23]. Students’ needs, prior knowledge, interests, and current understandings are paramount for student-centered teachers in facilitating student learning and guiding their students’ development [22]. One of the most effective ways to understand the mental space of learners is to get to know their mental images or metaphors. Through metaphorical thinking, one can understand learners’ motivations, the nature of errors, goals, and the nature of problems [24]. The term metaphor refers to something that is identified in the terms of another, in the way that the first thing receives the connotative and/or denotative meanings generally associated with the second one [25]. A metaphor is a conceptual tool for categorizing, organizing, thinking about, and ultimately shaping reality. Thus, metaphor underpins the way humans think [26]. Metaphor is related to exploratory behavior, mental imagery, visual and verbal processing, and creative thinking [24]. Metaphors can offer profound and imaginative ways of thinking [27]. They are often used when we are trying to find a way to express something difficult to express in words [28]. Metaphors highlight some aspects of experience while obscuring others [29]. They offer important opportunities to understand human unknowns and new situations [30]. As metaphors are largely used implicitly, they might help researchers to understand individuals’ implicit cognitions. At the same time, metaphors might be helpful for teachers or students themselves, because they can guide their reflection to unveil their implicit thoughts, and thus make them better teachers or learners [31]. Metaphor analysis can cast new light on familiar processes [32].

Considering the importance of the topic, it is appropriate to expand this aspect of ability in clinical training programs for students by creating new metaphors. Based on the literature review, no study was found that explains the metaphors of students in the field of mental disorders in Iran. Therefore, the present study aimed to identify nursing students’ metaphors for their first clinical experiences of encountering patients with mental disorders.

## Methods

### Study design and participants

This study was a descriptive qualitative study. Qualitative research is a form of social action that stresses on the way of people interpret, and make sense of their experiences to understand the social reality of individuals. It tries to help us to understand the social world in which we live, and why things are the way they are [33]. This type of research incorporates the recording, interpreting, and analyzing of non-numeric data with an attempt to uncover the deeper meanings of human experiences and behaviors [34]. Therefore, we were able to achieve the aim of this research only with a qualitative approach.

This study was conducted from April to September 2022. Participants were 18 undergraduate nursing students studying at Rasht and East Guilan nursing colleges of Guilan University of Medical Sciences who had “mental health nursing” clinical practicum experiences at Shafa Hospital in Rasht, the north of Iran. The average age of participants was 22 years. Eleven of them were girls and seven were boys. Purposeful sampling was used to collect data through semi-structured interviews. The inclusion criteria included studying at the undergraduate level of nursing, undergoing the psychiatric nursing clinical practicum, and willingness to participate in the study. The exclusion criterion was the unwillingness to continue participating in the study.

### Context of the study

This study was conducted at Guilan University of Medical Sciences in the north of Iran. This university has two nursing faculties in the cities of Rasht and Langeroud (east of Guilan province). The present study has targeted the “mental health nursing” practicum offered at the undergraduate level in the university. Nursing students have to pass two -credits of practicum at the same time as two -credits of “mental health nursing” course. The goals of the “mental health nursing” practicum include familiarization with psychiatric disorders, common treatments, and the nursing process in psychiatric patients. Nursing students go to Rasht Shafa Hospital for a “mental health nursing” practicum. Shafa Hospital is the largest psychiatric hospital in the north of Iran and the only psychiatric center at the University of Guilan province. It provides specialized psychiatry services and training of psychiatric assistants, and medical and paramedical students. This hospital is located on land with an area of 15,350 square meters and an infrastructure of 7,582 square meters. It has 248 beds and 6 wards. The wards include emergency, pediatrics, two women’s wards, and two men’s wards. It also is equipped with laboratory, radiology, encephalography, occupational therapy, ECT (Electroconvulsive therapy), and UROD (Ultra Rapid Opiate Detoxification) units.

The academic year at the Iranian universities is normally divided into two semesters. The “Mental health nursing” course and clinical practicum (practical course in mental health nursing) are offered in the second semester of the academic year for sixth-semester nursing students. The clinical practicum was conducted in such a way that at the beginning of the practicum period, an orientation meeting was held with the presence of 4 clinical professors and all nursing students of the 6th semester. In this meeting, the clinical professors explained to the students the educational objectives of the course, expectations, and how to deal with the patient. Then the students were divided into 4 groups of 5 to 6 people and each group went to the desired wards with a clinical professor. During the clinical practicum period, rotation was done so that all students had practical experience in all wards. Every clinical professor was present in the desired ward with the students the whole time. She first interviewed with a patient herself and the students observed how to deal with and interview the patient. Then other students were interviewed in the presence of the professor. Other cares were also performed in the presence of the clinical professor.

### Data collection

To conduct the research, the researcher first explained the purpose of the research to the participants and, if they wished, after obtaining their informed consent, interviewed them. Interviews were conducted individually and face-to-face using an interview guide. The time and place of the interviews were determined in coordination with the interviewees.

Before conducting the interview, the researcher built trust through communication methods. Data collection continued until data saturation. Saturation occurs when the collection and/or analysis of additional data adds nothing new to a piece of research [35]. In this study, saturation occurred when the collected data was a repetition of the previous data and no new codes were extracted. The data were saturated with 16 nursing students and two others were interviewed for more certainty.

After obtaining demographic information (such as age, gender, and field of study), the interview was conducted with open questions. The first question was: “Please tell us about your experience of a psychiatric nursing internship and the first encounter with a patient with a mental disorder.“, “How do you feel? “, and “What is this experience like and what is it likened to? You can mention any image that comes to your mind “. Next, questions such as the following were asked: “What is a psychiatric ward and hospital like? “, “What were the nurses of this hospital like? “, and “What was your clinical instructor like and what do you liken her to? “. The researcher tried to have minimal interference in the interview process. To guide the flow of the interview, the researcher asked leading and clarifying questions such as “Why do you compare yourself to…?” You may explain more.“, and “Do you mean…?” (Please see the supplementary file 1). The duration of each interview was 30–40 minutes and the total data collection lasted for six months.

There is no single way to do a metaphorical analysis; approaches range from detailed identification of metaphorical linguistic expressions to organizing data into metaphorical themes [32]. For data analysis in the present study, the Critical Metaphor Analysis approach was used. Metaphor analysis is a growing field of research and has particularly gained popularity in discourse and critical discourse studies over the last two decades [36]. Critical Metaphor Analysis (CMA) developed by Charteris-Black (2004) consists of three stages: identification of the metaphors, interpretation of the metaphors, and explanation of the metaphors [37, 38]. Identifying Metaphorical Expressions/Words is the first stage of Metaphor Identification which includes identifying words or groups of words that are used with a metaphoric sense or meaning via a close reading of the text [36]. This stage which also serves as an entry point in carrying out metaphoric analysis in a speech involves the determination of the kind of metaphors that occur in the text and their semantic correlation between the literal domain and the targeted metaphorical domain. The second stage of the Critical Metaphor Analysis which is the interpretation of metaphors also emphasizes ascertaining the kind of social relations that are enacted through the metaphors identified [38]. While ‘Metaphor Identification’ provides more general information regarding in-text features, metaphor interpretation, and explanation provide more specific in-depth information regarding out-of-text features which are ‘speech topics, context, and audience’ [36]. the third stage is the explanation of metaphors focuses on how metaphors interact within the context in which they occur [38]. To analyze the data, the text of the interviews was read several times. After studying all the descriptions of the participants and understanding their feelings, one code was assigned to each metaphor or important phrase. The codes were summarized and categorized according to their similarities and differences. Coding was done using MAXQDA 2007 software. It should be noted that there is no single answer as to what counts as a metaphor in the text; rather it is a matter of the researcher’s judgment and so a useful analysis strategy is to present and discuss findings with critical colleagues [32]. In this study, the first author conducted the initial analysis and then shared it with the second author. Following discussions, the analysis was restructured to focus on the five categories of experience described by the metaphors.

### Rigour

To guarantee trustworthiness as a balance to the imagination, coding should ideally be collaborative, with a portion of one person’s work being checked by another, and reflective [39]. To increase the study’s trustworthiness, two researchers analyzed the data, and then they agreed to compare their analysis. Also, two different experts analyzed the categories of metaphors developed in this study. Then, the categories made by experts and researchers were compared. After data analysis, discussions of the findings were made accordingly.

## Results

The analysis of students’ mental images related to their experience of dealing with mental illness led to the emergence of 36 metaphors and 5 categories. These categories were “the experience of dealing with a mental patient is similar to fear mixed with excitement”, “the patient is similar to an errant human”, " psychiatric hospital is similar to a prison”, “the nurse is similar to a prison guard”, and “the clinical instructor is similar to a supporter, sympathetic and knowledgeable friend”. These categories are presented in Table 1, and explained in detail below:

### Category 1: experience of dealing with a mental patient is similar to fear mixed with excitement

Patients suffering from mental illness may be threatening or violent, creating a feeling of insecurity for the students. In this category, students described the experience of dealing with mental patients as fear mixed with excitement and surprise. The participants’ metaphors indicated a scary experience for them. Because the behavior of patients with mental illness is unpredictable and could harm them. Being alone with the patient could have created a potentially dangerous situation by provoking threatening behavior in the patient. Therefore, on the one hand, nursing students were worried about communicating with the patient and were afraid of being alone with the patient. On the other hand, they wanted to communicate with them so that they might be able to help them. This dual feeling made them experience both fear and excitement. Some of the participants’ statements are below:

*I had a feeling of fear, which was removed early in the conversation with the patient. I was very excited during the entire internship because I thought that, unlike physical diseases, this disease has a kind of bitter story to listen to, and communication with these patients is always new*. (Student No. 6)

*I felt fear and stress because of the unusual behavior of the patients and I was excited about doing the interview*. (Student No. 4)

*I see each of them as a potential or actual killer and I have this feeling of fear towards them. Of course, I was not afraid when they communicated, looked kindly, or laughed. However, I was afraid of those who looked mysterious, were thinking, or had nervous tics and hand tremors* (Student No. 2).

*There was both excitement and fear. I tried not to show fear on my face and to deal with patients with a smile and a calm face*. (Student No. 8)

*I was interested in describing what they described and what they went through. I was more excited and curious*. (Student No. 11)

*I felt the same as when I went to the neonatal ward. People with different understandings than us. We were babies once and now we are different people. If we were in the same situation as them, maybe our work would reach there. It was interesting and exciting for me, like the pediatric ward*. (Student No. 1).

The fear of students was not only due to dealing with mental patients. Some students were afraid because they were not familiar with the environment and the atmosphere of the hospital. One of the students said:

*The most common feelings were fear and stress due to not being familiar with the environment. Of course, along with fear, there was also curiosity. I tried to show myself cool and calm*. (Student No. 3)

Some students also stated that they were just surprised and did not experience fear. One of the participants stated as follows:

*I was more surprised. The behavior they displayed and the behavior we saw during the interview were not the same. For example, one of the patients slept without clothes and put a knife on his head, but he behaved almost well*. (Student No. 5)

### Category 2: the patient is similar to an errant human

In this category, the students, considering the behavior of people with mental patients and their mentality, compared these patients to sinners. According to them, the patients are like those who are ignored and punished. One student said:

*The patients were like innocent people who were imprisoned and punished for no reason.* (Student No. 12)

The students felt compassion for the patients and expressed that no one understood them. They empathized with the patient and believed that patients with mental disorders should be treated with respect. One participant stated:

*Patients are like small children who are sometimes even mocked because of their thoughts and only their bodies have grown up and no one understands them*. (Student No. 15)

All people are entitled to receive the best mental health care available and be treated with humanity and respect. According to the statements of the students, it seems that this issue is not well respected in the mental hospital. Empathy is one of the essential skills that psychiatric nurses must develop. That a nurse can put him/herself in the patient’s place and have a correct understanding of his feelings. The statements of the participants in the present study indicated that they were able to empathize with the patients.

### Category 3: a psychiatric hospital is similar to a prison

The students in this study, considering the atmosphere of the hospital and its departments, compared it to a prison and a narrow and dark tunnel. High walls with metal doors with locks. The guards who constantly checked the entry and exit, the depressed clothes of the patients who compared them to a bat, all these things led them to a depressing and dark environment and affected the students’ morale. A distinctive feature of the students’ expressions was the sad ward and hospital, which reminded them of prison. Some of the students’ statements are below:

*The hospital had an atmosphere like a prison and it was bitter*. (Student No. 1)

*The environment is narrow, suffocating, and closed, and with a door that could be locked, it makes it look like a prison*. (Student No. 2)

*The environment of the hospital was like a prison. The doors of the rooms were only opened from the inside for security. The color of the hospital wall was depressing. The color of the patients’ clothes was not good. The patients did not have any special hobbies and most of them just walked in the corridor.* (Student No. 12)

*In my opinion, due to the old construction of the hospital, it looked like a prison. The stairs were dark and not well-lit. In general, this environment was not similar to normal hospitals.* (Student No. 17)

*From my point of view, the space can look like a prison because of the iron doors and the small part that is installed on the door to talk and communicate with the outside*. (Student No. 14)

*While forming a friendly relationship between them, the patients were very upset because the routine of life there was repetitive, fruitless, and suffocating. Like at the beginning of elementary school, when children have the anxiety of a legal and restrictive environment*. (Student No. 10)

*It is as if a series of bees were forced into a hive*. (Student No. 16)

### Category 4: the nurse is similar to a prison guard

Nursing staff in psychiatric practice should be aware that their attitudes might influence the quality of nursing care they deliver. According to the behavior of the staff towards the patients and with them, the students considered them dry and soulless people. Some of their statements are below:

*The nurses and guards of the mental ward were like prison guards. Of course, again, in my opinion, the nurses of this department were different from the nurses of other departments. They were quiet and did not smile.* (Student No. 13)

*The nurses seemed to me somewhat harsh and like a mother punishing her errant child*. (Student No. 14)

*Nurses are passive and like secretaries. They were only inside the nursing station because of the patient’s file*. (Student No. 10)

*We expected something else from the psychiatric nurses. Nurses seem to be afraid of patients and do not communicate much with patients*. (Student No. 2)

Nursing students stated that working in a mental hospital is very difficult and nurses who work in these hospitals should have a stronger personality than other nurses. One of the students said:

*In addition to the attractiveness and excitement of this different hospital, to work as a psychiatric nurse, one must have a stronger and more prepared personality*. (Student No. 9)

### Category 5: the clinical instructor is similar to a supporter, sympathetic, and knowledgeable friend

During the internship period, nursing students encounter different emotions and evaluate their adaptation skills, so that the initial assumptions of nursing students and their negative attitude towards psychiatric disorders are gradually changed, social distance is reduced, and empathy with patients increases. The role of the clinical instructor is very prominent in changing attitudes and creating adaptation. The students stated that the tutor is like a supporter and in her presence time, their fear is reduced and they can communicate with the patient more easily. This would lead them to change their attitude and feel empathetic towards the patient. One of the students said:

*It felt like being stuck among a tribe of dangerous people and not knowing what to do. Moreover, this feeling increased when we were in the corridor or the room without the tutor. With the presence of the clinical instructor, our fear was reduced and we could better communicate with the patient. This would change our attitude towards mental illness and make us empathize with the patient.* (Student No. 8)

In this category, students pointed to the scientific ability of clinical instructors, which increased their knowledge. Some of their statements are given below:

*The instructor was like a book. Because they were very skilled in the field of the theory of psychological topics, and it increased our awareness like a book* (Student No. 13).

*The tutor was like a mobile encyclopedia. Because he had a lot of information and knew the answers to all our questions* (Student No. 17).

In addition to the scientific ability of the clinical instructor, the students mentioned her compassion and kindness towards them. They also mentioned the clinical instructor’s communication skills, which had changed their attitudes towards mental illness. One participant stated:

*She (clinical instructor) was very kind and compassionate to us and tried to provide us with a calm atmosphere so that we would not be afraid.* (Student No. 13)

Another participant said:

*She also communicated very easily with patients. This made us learn from her and change our attitude towards mental illness.* (Student No. 15)

The participants in this research stated that during the internship process, their communication with patients became easier. They also stated that close contact with patients has changed their thinking about aggression and the impossibility of communicating with them. Some of them stated that they have gained a better understanding of mental health, the needs and expectations of patients, and their views on this disease and patients have changed in general.

**Table 1:** Categories emerged from nursing students’ metaphors from the first encounter with mental patients

Categories	Metaphors
Experience of dealing with a mental patient is similar to fear mixed with excitement	The feeling of fear, excitement, the bitter story to hear, stress, curiosity, surprise, and exciting
The patient is similar to an errant human	Prisoner, the potential or actual killer, mysterious look, punishment, lack of attention
A psychiatric hospital is similar to a prison	Bitter atmosphere, stuffy environment, locked doors, dull color of the walls, the bad color of clothes, lack of entertainment, old hospital, dark staircase, and iron doors with small hatches on them
The nurse is similar to a prison guard	taciturn, no smile, a mother who punishes her delinquent child, passive
The clinical instructor is similar to a supporter, sympathetic and knowledgeable friend	High theoretical knowledge, very skilled in the field of the theory, mobile encyclopedia, lots of information, answers to all questions, very kind and compassionate, easy communication with patients

## Discussion

This study aimed to identify nursing students’ metaphors from their first clinical experiences of dealing with patients with mental disorders. The analysis of nursing students’ metaphors led to the emergence of five categories. These categories included “experience of dealing with a mental patient is similar to fear mixed with excitement”, “patient is similar to an errant human”, " psychiatric hospital is similar to a prison”, “nurse is similar to a prison guard”, and “clinical instructor is similar to a supporter, sympathetic and knowledgeable friend”.

In the first category, under the title of “**experience of dealing with a mental patient is similar to fear mixed with excitement**”, most of the students had a sense of fear mixed with excitement. However, this fear decreased during the internship process and during the process of talking and dealing with these patients, and their views changed from fear to wonder and excitement. In a study conducted by Günaydin and Çoban (2021), all nursing students reported that they experienced negative emotions, especially during the first days of clinical training in mental health clinics. They stated that they felt fear, worry, anxiety, excitement, alienation, and loneliness. The reasons for these negative feelings were reported worry about saying something wrong, difficulty in making decisions about behavior, lack of knowledge, and the risk of encountering aggressive behavior [40]. In a study conducted by Başoğul (2021), most of the participating nursing students stated that the fear and discomfort they felt at the beginning of the clinical internship were reduced by getting to know the patients, and with time, they no longer thought that the patients were aggressive and dangerous. They also had a more advanced understanding of the importance of stigma. In addition, they stated that there was a change in their view of patients and diseases [20]. One study also showed that with time on a clinical internship, students considered people with mental disorders to be less dangerous [41]. Other studies have also revealed negative attitudes toward mental illness among nursing students [42, 43]. Nevertheless, in several studies that have evaluated nursing students’ beliefs about mental illnesses, it has been reported that they have positive beliefs about mental illnesses [44, 45]. However, having negative feelings and attitudes can affect the improvement of students’ clinical skills and may cause them to avoid choosing the profession of mental health nursing when planning their future careers [46]. On the other hand, when healthcare professionals have positive beliefs about mental illness, this view can have a positive effect on patient and family support during the treatment process [45].

Nursing students are exposed to demands, complicated care, and patients during their mental internship where they face their emotions and evaluate their adaptive skills so that the initial preconceptions of nursing students and their negative attitudes toward psychiatric disorders are gradually changed, social distance is reduced and empathy toward patients is promoted [47]. The clinical experience of direct contact and familiarity with psychiatric disorders in a clinical setting by combining new information and challenging negative beliefs reduce the anxiety associated with exposure to these patients which can help to improve empathy in students [48]. The findings of the present study were similar to the results of most of the studies in the relevant literature. However, the results in the present study were inconsistent with some other studies. This difference in the findings can be related to the characteristics of the clinical setting, duration of clinical practicum, and factors related to students.

In the second category, under the title of “**patient is similar to an errant human**”, according to the way other people treat these patients, the students compared them to sinful person who is being punished. In different studies, different interpretations of a patient with a mental disorder have been mentioned. Students in the study conducted by Başoğul (2021) also stated that these patients are marginalized, ridiculed, and treated as if they are different from others. These students used words such as confinement in society, difficulty, difference, unluckiness, lostness, derangement, obscurity, and innocence to explain mental illness [20]. Some studies have also used the metaphor of “a fragmented entity” to describe patients with depression [49] and schizophrenia [50]. The difference in the metaphors of the patient with a mental disorder can be due to the multidimensional and complex nature of the mental illness. However, people with mental disorders have often experienced high levels of abuse or neglect of human rights, including the rights to liberty and treatment [51]. As humans, we share each other’s worlds and thus become responsible for each other’s trust as an ethical demand [52].

Students in the present study empathized with the patient and believed that patients with mental disorders should be treated with respect. The ultimate aim of caring in professional nursing is described as preserving the patient’s dignity, absolute value as a human being, and his/her right to self- determination [52]. Taking time to listen to the patient, which includes empathy, silence, attention to both verbal and nonverbal communication, and the ability to be non-judgmental and accepting, has always been considered a crucial component of nursing care [53]. Empathy, as a backbone of therapeutic relationships, enables healthcare providers to accurately elicit and identify patient preferences and values in response to health problems, and thus, improves patient health outcomes. Furthermore, empathy is a cognitive attribute a way of recognizing and conveying understanding of patients’ concerns that allow them to feel respect, comfort, and support and may improve the quality of patient care [54]. In an encounter with a patient with mental illness, both parties need the courage to enter into a conversation. It requires reciprocal trust, earlier described as a core component in a caring relationship. The nurse has to trust the patient and vice versa [53]. Being able to put himself or herself in the patient’s shoes does not mean that the nurse has had the same experiences as the patient, i.e., being sympathized. Nevertheless, by listening and sensing the importance of the situation to the patient, the nurse can imagine the patient’s feelings about the experience [55].

In the third category, under the title of “**psychiatric hospital is similar to a prison**”, the students compared the hospital and its departments to a prison. There is documentary evidence from many parts of the world that people with psychiatric disorders experience the most severe cases of human rights violations, including being tied down to the bed, kept isolated in psychiatric hospitals, chained and imprisoned in small cells, and mistreatment [56]. Moreover, despite the shortage of hospital beds and the need for many patients to be admitted, they are sometimes admitted with a violation of their rights [57]. In a study conducted by Heydari et al. (2019), the main theme “Psychiatric hospital: an unsafe place” with the sub-theme “an egregious hospital” shows that psychiatric hospitals are in poor conditions. Their buildings are old and are often located outside the city. In addition, patients in these hospitals face poor conditions of clothing, nutrition, and health [56]. In a study conducted by Möller et al. (2016), people’s perception of psychiatric hospitals included locked doors, restrictive clothing, psychotropic drugs that are addictive, and invasive and ineffective treatment, while their positive effects were underestimated [58]. Patients with psychiatric dysfunction in the study conducted by Abbasi et al. (2010) felt that they were treated with disrespect and mocked in the mental health systems [57]. Nursing students participating in Başoğul’s study (2021) suggested creating a treatment environment in which, in addition to other services, mental health services are also provided, protecting patients’ rights and amending legal regulations [20]. World Health Organization (WHO) highlights the urgent need to transform mental health and mental health care. The report of this organization urges mental health decision-makers and advocates to step up commitment and action to change attitudes, actions, and approaches to mental health, its determinants, and mental health care [59].

In the fourth category, under the title of “**nurse is similar to the prison guard**”, students named the nurses working in the psychiatric ward as dry, soulless, and afraid of patients. In the study conducted by Coll-Florit et al. (2021), metaphors for the mental health care professional team were named in three domains under the headings Journey, War, and Power [2]. In the study by Heydari et al. (2019), the sub-theme of “cold-hearted white collars” showed the mistreatment and inhuman behavior of hospital staff towards patients who ignore the rights of clients [56].

Mental health nurses, as the largest health-care professional team, provide the most direct care for individuals with mental illness and their families [54]. The nurses are expected to offer professional care and assess each patient’s condition while showing respect for the patient’s self-determination and integrity. However, nurses have been found to dichotomize medical care and a caring relationship, with their primary focus being on medical care and treatment [53]. It is likely that mental healthcare professionals working in inpatient psychiatric units tend to view individuals with mental illnesses as more dangerous and desire less interaction with these individuals [54]. This attitude can affect their encounters with patients with mental illness who do not manifest explicit emergency somatic symptoms [53]. Nurses may experience a complex contradiction in their professional role, as a willingness to reach a patient that is not perceived as interesting compared with others [52]. Healthcare providers, both in the community and in hospitals, have an opportunity to influence patients. As a result, overt negative attitudes when interacting with the patient cause anxiety or discomfort and lead to ineffective counseling or lack of medical care. Unfortunately, mental health centers use a humiliating approach to identifying patients, which distorts their identity [56]. Meanwhile, according to the opinion of many nursing students in the study conducted by Günaydin and Çoban (2021), nursing personnel should play an active role in the clinical education of students. They should act as role models for nursing students, based on the findings of this study, the educational roles of nurses in mental health clinics were insufficient and very weak and they were not even able to communicate with the students and, according to the students, even noticed their existence if they had not been there [40]. Some nursing students participating in the Başoğul (2021) study stated that improving the communication skills of health workers and focusing on patient-centered care could be effective in reducing stigma. In addition, they emphasized the importance of nurses’ expertise in this field [20].

In the fifth category, under the title of “cl**inical instructor is similar to a supporter, sympathetic and knowledgeable friend**”, students stated that the behavior of the clinical instructors with them and with the patients made them better able to communicate with the patients and showed more empathy. This confirms the value of the role model of a skilled clinical instructor who could guide nursing students and train them in an empathetic approach [60]. Nursing education and training programs should aim to equip mental health nurses with empathic reactions and positive attitudes in the care of individuals suffering from mental illnesses [54]. With proper psychiatric nursing education, empathetic skills can prepare students and future nurses for delivering emotionally competent nursing practice, facilitating communication, and creating positive changes in clinical environments [60]. The clinical instructor may act as a role model while showing acceptance and emotional understanding to patients and demonstrating higher levels of empathy within a caring professional nurse-patient relationship [55]. In general, the role of mental health educators and nurses in preventing negative beliefs and attitudes about mental health clinical practice is vital for nursing students. From the point of view of nursing students, the clinical instructor plays the most active role in helping them learn in a clinical environment [40]. In addition, nursing educators can guide students in redesigning the curriculum to develop methods that can facilitate competence and confidence to reduce students’ prejudice, fear, and stress in clinical practice [61]. However, academic training in this area should be designed to help change attitudes, which includes greater use of educational strategies that challenge beliefs and assumptions and promote a commitment to providing comprehensive care for people with mental illness [2].

However, the beliefs and attitudes on mental illnesses can cause individuals to experience problems in support, treatment, and rehabilitation processes [3]. How people with mental disorders are viewed by treatment providers can have a significant impact on treatment outcomes and their quality of life [62]. When healthcare professionals have positive beliefs about mental illnesses, this can have a positive effect on the support of the patient and the family during the treatment process [3]. Metaphors can create a bridge between subjective experience and clinical descriptions [28]. They show how we perceive reality [63]. They allow us to form concepts to define reality for ourselves and to describe our experiences to others [28]. The emerged themes through the metaphors created by the nursing students can serve to guide the development of educational content aimed at reducing the prejudices negative beliefs and attitudes toward individuals with mental problems to ensure that nurses can work more effectively with this group in the future [3].

## Limitations

The main limitation of the present study was that it was conducted on undergraduate nursing students of the Guilan University of Medical Sciences. Therefore, the generalization of the findings is limited due to the nature of qualitative studies and differences in contexts and cultures. It is suggested that future research among students of other disciplines in different fields and cultures be done as quantitative, qualitative, or mixed research.

## Conclusion

Metaphors allow problems about which there is insufficient knowledge or issues that are not well understood to be easily understood and to find a deeper meaning. The perceptions of nursing students, who deal with people suffering from mental health problems and who may directly provide care for these people in the future are a factor that may affect the quality of care. In this study, nursing students who experienced their first encounter with a patient with mental disorders during the psychiatric nursing internship participated. They described this experience; the patient, the psychiatric hospital, the nurses working in the psychiatric centers, and the clinical instructors of this field with words and metaphors. Metaphors of nursing students showed a negative attitude towards the psychiatric hospital and health care providers for these patients.

It is suggested that to change attitudes and prevent stigmatization of these patients, psychiatric hospitals should be established in government hospitals, and nursing care providers in these centers should also learn how to deal with mental illness and have more appropriate interaction, along with support and acceptance. It is also suggested that the findings of this study should be taken into consideration in planning the clinical education of nursing students, and nursing educators should use the results of the study to guide nursing students who wish to work in this field in the future, as well as in curriculum development.

### Electronic supplementary material

Below is the link to the electronic supplementary material.

**Supplementary Material 1:** Interview guide questions

## Data Availability

The data that support the findings of this study are available upon reasonable request.
