# Supplementary material for: Nursing students’ metaphors of first clinical experiences of encountering patients with mental disorders
Source: BMC Nurs. 2024 Feb 4;23:95. doi: 10.1186/s12912-024-01780-9 (PMC10838411; doi:10.1186/s12912-024-01780-9)
Supplement: Supplementary file 1 — Supplementary Material 1: Interview guide questions [file 12912_2024_1780_MOESM1_ESM.docx]

**Interview guide questions**

| 1 | Please tell me about your feelings about the psychiatric nursing internship and the first encounter with a patient with mental disorder. How do you feel? |
| --- | --- |
| 2 | What is this experience like and what is it likened to? You can mention any image that comes to your mind. |
| 3 | What is your understanding of a patient with mental disorders? |
| 4 | What do you think about caring for a patient with mental disorders? |
| 5 | What is a psychiatric ward and hospital like? Please explain why you liken it to...? |
| 6 | What were the nurses of this hospital like? Please explain why you liken it to...? |
| 7 | What do you think about your future career as a mental health nurse? |
| 8 | What was your clinical instructor like and what do you liken her to? Please explain why you liken it to...? |
